# Influence of Excessively High Temperatures on the Fruit Growth and Physicochemical Properties of Shiranuhi Mandarin in Plastic-Film Greenhouse Cultivation

**DOI:** 10.3390/plants10081525

**Published:** 2021-07-26

**Authors:** Misun Kim, Sock-Beom Kang, Seok Kyu Yun, Sang Suk Kim, Jaeho Joa, YoSup Park

**Affiliations:** Citrus Research Institute, National Institute of Horticultural & Herbal Science, RDA, Jeju 63607, Korea; mkim2019@korea.kr (M.K.); hortkang@korea.kr (S.-B.K.); sky0611@korea.kr (S.K.Y.); sskim0626@korea.kr (S.S.K.); choa0313@korea.kr (J.J.)

**Keywords:** excessively high temperature, amino acids, antioxidant activity, citrus

## Abstract

Excessively high temperatures frequently occur between spring and late fall because of global warming. These problems have a negative effect on fruit production capacity and actual production. However, there is a lack of information on the effects of excessively high temperatures (exceeding the optimum range) on the growth of citrus fruits. Thus, the objective of this present study was to determine the effects of excessively high temperatures on Shiranuhi mandarin fruit growth, physiological characteristics, amino acid contents, and antioxidant activity. In this study, five different high-temperature treatments were applied: normal atmospheric temperature (Con), Con + 4 °C during the day (D4), Con + 8 °C during the day (D8), Con + 4 °C during the night (N4), and Con + 4 °C during the day and night (DN4). The total soluble sugar contents were lowest in D8, and the acid content under DN4 was significantly lower than that under Con. Rising temperature during the day or night affected the colouration of the rind, and the free sugar composition ratio under D8 was lower than those under the other treatments. The total amino acid content tended to increase with increasing temperature during the day. The total flavonoid and total phenolic contents in the peel were significantly higher under Con and N4 than the other treatments and in the pulp under Con.

## 1. Introduction

Shiranuhi mandarin [(*Citrus unshiu* × *C. sinensis*) × *C. reticulata*], a late-harvest cultivar developed in Japan, was introduced to Korea in the 1980s. This cultivar is the most popular late-harvest cultivar in Korea on the basis of cost-benefit analysis. Thus, the Shiranuhi mandarin cultivation area is increasing not only in Jeju, the main production area, but also along the southern coast. Compared with Satuma mandarin (*C. unshiu*) growth, Shiranuhi mandarin growth requires warmer temperatures; as such, the latter is grown mainly in plastic-film greenhouses, but is also cultivated in open fields in some warm areas of Jeju [1].

According to the Fifth Assessment Report presented by the Intergovernmental Panel on Climate Change (IPCC), a long-term increase in the global mean temperature is predicted [2]. On the Korean Peninsula under scenario RCP8.5, the average temperature is predicted to increase by 0.4 °C every 10 years and is expected to increase by 4.4 °C over 940 ppm CO_2_ in 2100 [3]. By applying a climate change scenario, Moon et al. [1] studied the possible effects on the cultivation of Satuma and Shiranuhi mandarin. The authors predicted that the cultivation of Satuma mandarins would decrease, while the cultivation of late-harvest cultivars such as Shiranuhi mandarins would increase. However, high temperatures and drought due to climate change are expected to cause difficulties in citrus production [4]. Additionally, abnormal weather conditions are frequent worldwide, and high temperatures have occurred in Korea throughout the past decade from spring to summer and even during late autumn [3]. High temperature induces changes in membrane-based processes such as photosynthesis and respiration, resulting in a generation of reactive oxygen species [5,6]. In citruses, high temperatures cause a decrease in chlorophyll, an accumulation of carotenoids, and delayed colouration. Soluble solids also accumulate to high levels, the phenomenon of which decreases the acidity content and storage quality [7].

The composition of secondary metabolites in grapefruits (*C. paradise*) is affected by the cultivation environment and harvest time [8]. Additionally, heat treatment affects phenolic compounds and the antioxidant capacity of citrus peel extracts [9].

Therefore, our study investigated the changes in physicochemical properties, amino acids, and antioxidant activity affecting Shiranuhi mandarin quality caused by excessive temperatures during the day and night. The results can be used as basic data for the effects of excessively high temperatures in environments in the future.

## 2. Results

### 2.1. Effects of Excessively High Temperatures on Shoot Growth and Fruit Quality

**Figure 1:**
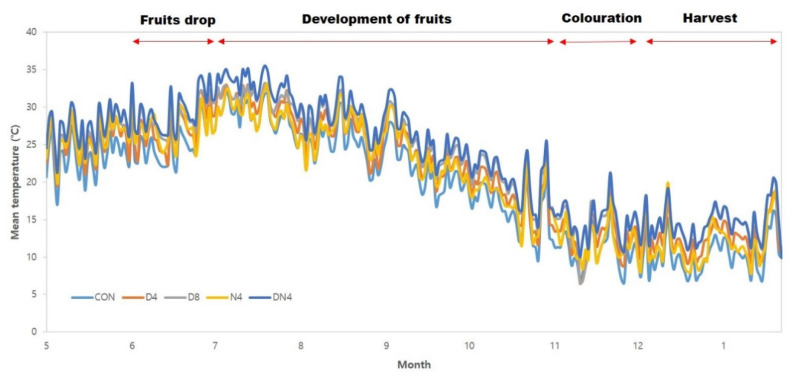
Change in daily mean air temperature in a plastic-film greenhouse with excessively high temperatures.

The average daily air temperature during the Shiranuhi mandarin growth period is shown in Figure 1. The average air temperature of the normal atmospheric temperature (Con) treatment during the fruit development period (from July to November) was 23 °C, which was 10.5 °C during the fruit harvest period (December to February). The high-temperature treatments were 1.7 °C to 4.1 °C higher in temperature than the Con treatment during the experiment period. Additionally, the average daily temperature range during the experiment period was 11.7 °C in the Con treatment, 15.7 °C in the Con + 4 °C during the day (D4) treatment, 18.9 °C in the Con + 8 °C during the day (D8) treatment, 10.6 °C in the Con + 4 °C during the night (N4) and 11.9 °C in the Con + 4 °C during the day and night (DN4) treatment. The accumulation times of temperatures greater than 30 °C during the experiment were 123 h, 1002 h, 1543 h, 537 h, and 1224 h in the Con, D4, D8, N4, and DN4 treatments, respectively, indicating greater accumulation under the temperature treatments than under the Con treatment (data not shown).

**Figure 2:**
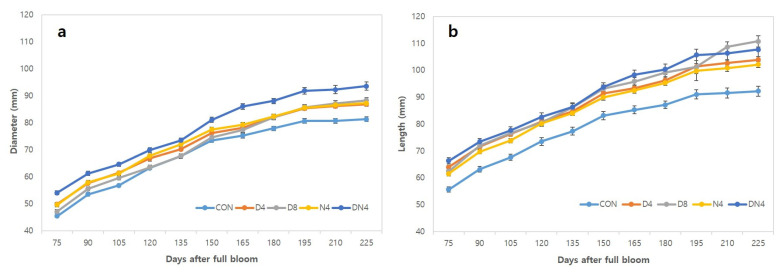
Shiranuhi mandarin fruit growth increased during development. (**a**) Diameter (mm). (**b**) Length (mm). Error bars represent standard errors (*n* = 20).

Figure 2 shows the growth pattern of Shiranuhi mandarin fruit according to daytime/night-time high-temperature treatments. All of the treatments markedly increased the growth from the initial stage to the fruit-development stage, after which growth slowed during the transition to the maturity stage, reflecting a typical single sigmoidal growth curve. Compared with the Con treatment, the high-temperature treatments resulted in more vigorous fruit development, and among the treatments, the DN4 treatment resulted in the largest transverse and longitudinal fruit diameters.

The shoot growth parameters and fruit set of Shiranuhi mandarin trees under daytime/night-time high-temperature treatments are shown in Table 1. The leaf/fruit ratio was approximately 100 leaves/1 fruit, which was similar between treatments. The number of shoots per branch ranged from 0.3 to 0.9, with some variation depending on the temperature. Noticeably, the number of shoots was 1.3-fold increased under the D8 treatment and 2.3-fold decreased under the N4 treatment compared to that under the Con treatment. The shoot length under the N4 treatment increased approximately 1.7-fold compared to that under the Con treatment; the trees under the N4 treatment displayed the most vigorous growth. The fruit set increased approximately 1.3-fold under the N4 treatment compared to the Con treatment, but decreased approximately 1.9-fold under the D8 and DN4 treatments. Therefore, the increased temperature during the day (D4 and D8) and day/night (N4) caused a decrease in the fruit set. After the final harvest, the trees under the Con treatment presented the highest number of fruits and a total fruit weight of 130 fruits/tree and 25.9 kg/tree, respectively. Under the D8 treatment, the number of fruits and total fruit weight decreased by 4.4-fold and 3.4-fold, respectively, compared to those under the Con treatment. After measuring the fresh weight of the branches by uniformly pruning them after harvest, the D8 treatment resulted in the highest fresh weight (8.2 kg), whereas the DN4 treatment resulted in the lowest fresh weight (3.7 kg).

**Figure 3:**
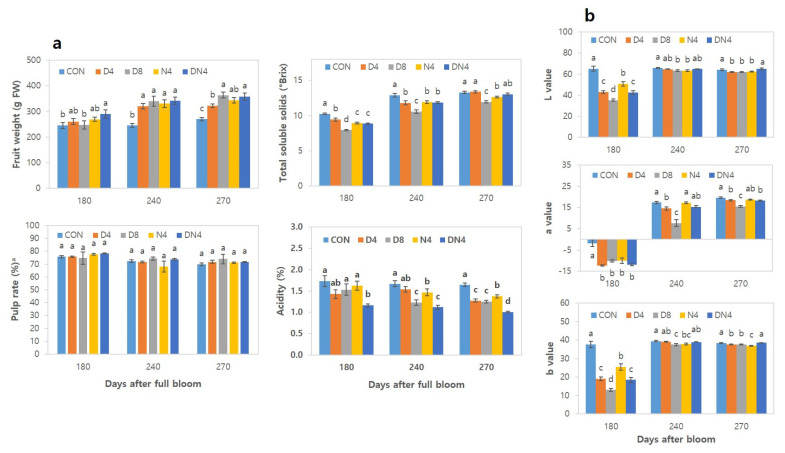
Effects of excessively high temperatures on the characteristics of fruits of Shiranuhi mandarin trees grown in plastic-film greenhouses. (**a**) Fruit weight, pulp rate, total soluble solids, and acidity. (**b**) Colorimetric value. ^a^ Pulp rate is the percentage of pulp weight per total fruit weight. The means followed by different letters within columns are significantly different according to Duncan’s Multiple Range Test (*p* < 0.05).

The effects of high temperatures on fruit weight, pulp rate, TSS content, acidity, and colouration are summarized in Figure 3. The fruit weight increased with increasing days after full bloom (DAFB): those under the Con treatment were 270 g at harvest (270 DAFB), whereas those under the D8 treatment were the highest (363 g). There was no significant difference in pulp rate between the treatments. The TSS content of the fruits increased with increasing DAFB, and those under the Con treatment presented the highest value—10.2°Brix at 180 DAFB. In the harvest period at 270 DAFB, the fruits of the trees under the Con and D4 treatments presented the highest total soluble solids (TSS) content of 13.3° Brix, whereas those under the D8 treatment presented the lowest content of 11.9° Brix. The TSS content was low in the early stage of colouration under the DN4 treatment, but the TSS dramatically increased 13.1° Brix by harvest, which was similar to that under the Con treatment. The acidity content decreased with increasing DAFB and was lower under the high-temperature treatments than under the Con treatment. At 180 DAFB, the acidity content was highest under the Con treatment (1.7%), and the fruits under the DN4 treatment showed a remarkably low acid content of 1.2%. This result was similar to the trend observed at 270 DAFB. The ranking based on the sugar/acid ratio of fruits of the treatments was as follows: DN4 (13/1) > D4 (11/1) > D8 (10/1) > N4 (9/1) > CON (8/1).

Colorimetric analysis via a chromameter of the pericarp (Figure 3b) revealed that the *L* value of the fruits was the greatest under the Con treatment at 180 DAFB, while the fruits under the D8 treatment presented the lowest *L* value. A significant difference between the treatments was found. The *a* value (red or green) was significantly lower at 180 DAFB, indicating greener colour, under all the treatments except the Con treatment. The *a* values at 270 DAFB (harvest) were highest under the Con treatments, whereas the lowest value was observed under the D8 treatment. The *b* value (yellow or blue) significantly differed between treatments at 180 DAFB but significantly decreased as harvest time approached.

Table 2 shows the results of the analysis of the transverse and vertical diameters of the prominent bump on the top of the fruit, the thickness of the peel, and firmness. The prominent bump on the top Shiranuhi mandarin fruit is a cultivar-specific characteristic that is an important determinant of the quality. The large prominent bump on the top of fruits under the D8 treatment was 44.1mm in diameter and 27.2 mm in length, which significantly differed from that under the other treatments. The peel was thicker under the D8 and N4 treatments than under the other treatments, but there was no significant difference between treatments. The fruits were firmest under the D8 treatment, and those under the DN4 treatment were the softest (8.6 N).

**Figure 4:**
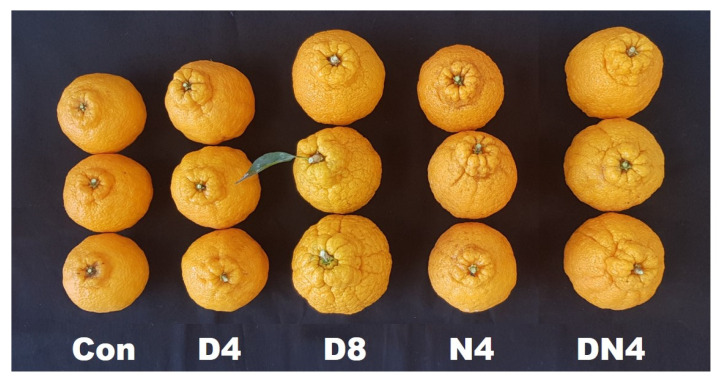
Effect of excessively high temperatures on the fruit of Shiranuhi mandarin trees grown in a plastic-film greenhouse.

The phenotypes of the Shiranuhi mandarin fruits at harvest are shown in Figure 4. Compared with the Con treatment, the D8 treatment resulted in a more prominent bump on the top of the fruit, as reflected in Table 2. Additionally, the fruit rind had a fairly rough shape.

### 2.2. Effects of Excessively High Temperatures on Fruit Taste

**Figure 5:**
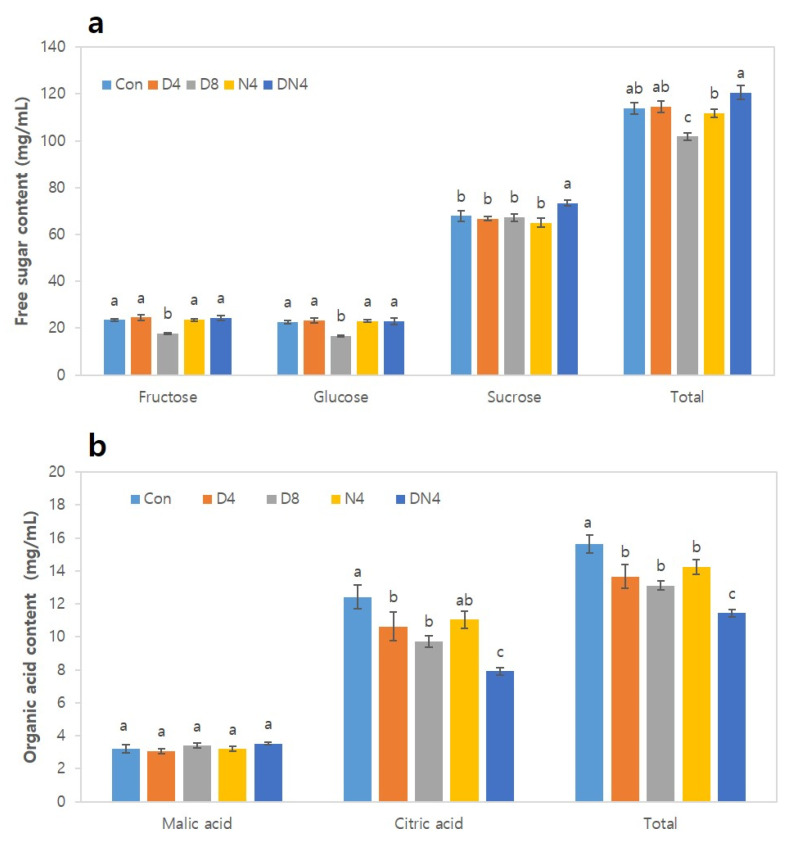
Effects of excessively high temperatures on the free sugar and organic acid contents of the fruit of Shiranuhi mandarin trees grown in a plastic-film greenhouse. (**a**) Free sugar content. (**b**) Organic acid content. The data are the means ± SEs (*n* = 12). The means followed by different letters within columns are significantly different according to Duncan’s Multiple Range Test (*p* < 0.05).

The effects of high temperatures on free sugar and organic acid contents in the fruits after harvest were analysed (Figure 5). The sucrose content accounted for approximately 60% of the total free sugar content, although there were some differences depending on the treatment. Fructose and glucose were significantly lower (17.7 mg/mL and 16.8 mg/mL, respectively) under the D8 treatment than under the other treatments. On the other hand, sucrose was highest under the DN4 treatment, at 73.4 mg/mL, but similar under the other treatments. The total free sugar content under the DN4 treatment was 120.6 mg/mL, which was approximately 1.1-fold higher than that under the Con treatment (114.0 mg/mL). However, the total free sugar content under the D8 treatment was approximately 1.1-fold lower (101.8 mg) than that under the Con treatment (Figure 5a). Analysis of the main organic acids of citrus fruits, such as citric acid, malic acid, and oxalic acid, revealed that citric acid accounted for more than 85% of the total organic acid content (Figure 5b), and a very small amount of oxalic acid was detected. The citric acid content was the lowest under the DN4 treatment (7.9 mg/mL), while that under the Con treatment was the highest (12.4 mg/mL), indicating a significant difference between treatments. However, there was no significant difference in malic acid content between any of the treatments.

The ranking of the total amino acid contents based on the 20 kinds of amino acids analysed after harvest was as follows: D8 (1276 µg) > D4 (1113 µg) > DN4 (920 µg) > CON (888 µg) > N4 (869) µg (Table 3). Arginine (Arg), aspartate (Asp), and asparagine (Asn) in the fruits under the Con treatment accounted for 60.9% of the total amino acids—28.1%, 16.5%, and 16.3%, respectively. These three main amino acids also accounted for approximately 60% of the amino acids in the other treatments. Compared with the other treatments, the D8 treatment resulted in relatively high contents of glycine (Gly), serine (Ser), Asn, Asp, and glutamine (Gln).

By classifying the flavour according to the methods of Kawai [10], we divided the samples into five groups: (1) sweetness, (2) bitterness, (3) sour and umami, (4) sweetness and bitterness, and (5) others (Table 4). The taste ratio to total amino acid under the Con treatment was 26% sweetness, 30% sweetness/bitterness, 40% sour/umami, and 4% bitterness. However, under the D8 treatment, the sweetness decreased by 2%, sweetness and bitterness decreased by 3%, the bitterness flavour decreased by 1%, and the sour/umami increased by 6%, compared to those under the Con treatment. Compared with the Con treatment, the DN4 treatment decreased the sweetness by 4%, the sweetness and bitterness by 11%, while the bitterness increased by 3%. In particular, the sour and umami flavour significantly increased by 12% compared with that under the Con treatment. Consequently, the sweetness, bitterness, and sour/umami flavour related amino acids were stronger under the D8, DN4, and D8 treatments, respectively, than under the other treatments, but there was no difference in sweetness/bitterness.

### 2.3. Effects of Excessively High Temperatures on Flavonoid Contents and Antioxidant Activities

We evaluated the relationship between flavonoid contents and high temperature for ten main citrus flavonoids (Table 5). A total of five flavonoids, rutin, narirutin, hesperidin, nobiletin, and tangeretin, were detected in the peel. However, only three flavonoids, rutin, narirutin, and hesperidin, were identified in the pulp. The total flavonoid content in the peel was the highest (approximately 3009 mg/100 g) under the Con treatment and lowest under the D4 treatment (2387 mg/100 g). Compared with those under the other treatments, the fruit peels under the N4 treatment had significantly higher narirutin and tangeretin contents (883 mg/100 g and 32 mg/100 g, respectively). The total flavonoid content in the pulp was the highest, at approximately 576 mg/100 g under the Con treatment, and lowest under the D8 treatment, at 376 mg/100 g. Narirutin and hesperidin were the highest under the Con treatment, at 253 mg/100 g and 277 mg/100 g, respectively, which were significantly different.

Table 6 Shows the results for the total phenolic content (TPC) and antioxidant activity. The TPC ranged from 3365 mg/100 g to 3931 mg/100 g in the peel and 1074 mg/100 g to 1438 mg/100 g in the pulp, which significantly differed depending on the part of the fruit and temperature treatment. In the peel extracts, the TPC was significantly higher under the Con and N4 treatments and lowest under the D4 treatment. Among the TPCs in the pulp extracts, those under the Con treatment were the greatest and those under the D8 treatment were the lowest. The IC50 value (the concentration of antioxidants needed to decrease the initial concentration by 50%) of 1,1-diphenyl-2-picrylhydrazyl (DPPH) in the peel extracts was highest under the D4 and D8 treatments, while in the pulp extracts, it was highest under the D4, D8, and DN4 treatments. The IC50 value of 2,2′-azinobis (3-ethylbenzothiazoline-6-sulfonic acid) (ABTS) in the peel extracts was also the highest under the D4 treatments, but there was no difference in the pulp extracts. 

## 3. Discussion

### 3.1. Effects of Excessively High Temperatures on Shoot Growth and Fruit Quality

In citruses, under the high temperatures prevailing in the tropics, citrus fruit development is fast, and the fruits become very large. The heat unit requirements for the maturation of Valencia oranges grown in cool regions is twice that of Valencia oranges grown in tropical regions [11]. In the present study, an increase in the daytime and/or night-time temperature also promoted increases in the transverse and longitudinal diameters of the fruits, and a large difference in longitudinal diameter was observed (Figure 2b). Moon et al. [12] similarly reported vigorous enlargement of Shiranuhi mandarin fruits through spring heating treatment compared to a control treatment. The author also observed that the increase in longitudinal diameter was more vigorous than that of the transverse diameter. Therefore, it seems that high temperature at the fruit development stage is closely related to fruit enlargement.

In common mandarins such as those of *C. unshiu*, the neck at the fruit stem end reduces market value because of its poor appearance. However, this is an inherent characteristic of the cultivar Shiranuhi, and it acts as an important factor in determining market value. There was a strong correlation between the longitudinal diameter of the fruit and the length of the neck of the fruit (0.67, *p* < 0.01; data not shown). High temperature also seems to be a factor that promotes neck development at the fruit stem. Excessively high temperatures during the day seemingly have a greater effect on the neck of the fruit stem. Moreover, excessively high temperatures during the day cause a high daily temperature range, which appears to have a greater influence on the neck of the fruit stem.

High-temperature treatments in plastic houses resulted in pummelo (*C. grandis* L. Osbeck) fruits with pyriform shapes and prominent necks at the stem end, and these fruits had the shortest transverse/longitudinal diameters [13]. In the present study, the number of shoots and shoot length under the D8 treatment were greater than those under the other treatments. This was the result of an increase in vegetative growth (fresh weight) as the fruit set decreased (Table 1). On the other hand, the N4 treatment presented the lowest number of shoots; however, the fruit set was higher than those under the other treatments, and the yield was lower than those under the Con and D4 treatments. The most significant factors for heat stress-related yield loss for cereals include a shortened developmental phase, reduced light perception, and changes in the processes associated with transpiration, photosynthesis, and respiration [5,14].

Continuous fruit decay occurred in the trees after the colouration period, and the D8 treatment resulted in 2.5-fold more continuous fruit decay than the Con treatment did (data not shown). This difference is thought to have been caused by the generation of dew on the pericarp caused by heat from the ground. The TSS/total acidity ratio is a physiological parameter that can determine fruit quality. The standard for Shiranuhi mandarin fruit quality in Korea is greater than 12° Brix for the TSS content and 1.1% acidity. In our study, a markedly increased daytime temperature (D8) reduced the accumulation of sugars, while an increased daytime/night-time temperature decreased the acidity. In particular, days and nights with increased temperatures seemingly had a greater effect on acid accumulation than days or nights with normal temperature alone. Previous studies have shown reduced TSS contents and citric acid contents in citrus fruits under high-temperature treatment [12,13]. Hutton and Landsberg [15] predicted a decrease in TSS and acidity with a rise in temperature sums over time (effective heat units) during orange fruit development. In kiwifruit, heating during the starch accumulation period significantly reduced the contents of carbohydrates, while heating during the fruit maturation period reduced the starch content and delayed fruit maturity, showing different trends according to heating duration [16]. Grigenberger et al. [17] proposed that elevated temperature leads to increased rates of respiration, resulting in a decline in 3-phosphoglyceric acid, which then inhibits enzyme and starch synthesis in potato tubers, indicating reduced starch contents. Moon et al. [12] also reported that the photosynthesis rate, stomatal conductance, and transpiration rate of citrus leaves during a spring heating treatment were much higher than those under a control treatment. Stress treatments, such as high temperature, drought, and high temperature/drought stress, caused reduced sucrose and starch contents in wheat grain [18]. The authors found that with the stress treatments, key enzyme activities and the related gene expression led to not only repressed conversion of sucrose to starch, but also a decrease in sucrose content. Therefore, it is thought that the increase in respiration rate due to high temperatures interferes with the transfer of photo-assimilates from leaves to fruits, and analysis of enzymes associated with sugar or acid synthesis or related gene-level studies are needed to confirm this phenomenon.

Pericarp colour is another important factor that determines fruit quality. The pigmentation of mandarin and oranges varies greatly among species, and the two main types of isoprenoid-derived pigments (chlorophylls and carotenoids) are responsible for citrus fruit colouration [19]. When a citrus fruit matures, the amount of chlorophyll decreases, and carotenoids accumulate. Spiegel-Roy and Goldschmidt [11] reported that the decrease in chlorophyll in citrus rinds coincides almost with the onset of carotenoid accumulation. With respect to the colour of Shiranuhi mandarin pericarps in this study, the difference between the *L* and *b* values decreased considerably at harvest time, but the *a* value clearly differed between the treatments (Figure 3b). The increased temperature during the day seemed to have a negative effect on fruit colouration. Itle and Kabelka [20] suggested that the L* colour value correlated negatively with lutein and total carotenoid contents, whereas the a* and b* colour values were strongly correlated with total carotenoids and lutein, respectively. The authors suggested that a negative correlation between L* and certain carotenoids would be expected, because any increase in pigment content would increase darkness and thereby decrease the L*. Matsumto [21] also reported that the expression of carotenoid biosynthesis-related genes in citrus fruit are sensitive to temperature. In subtropical areas, pigmentation occurs at night-time temperatures of 8 to 15 °C and 20 °C. Moderate–low outdoor temperatures stimulate the accumulation of *β,β*-xanthophylls and C_30_ apocarotenoids, which are responsible for the orange colour of citrus fruits and the expression of carotenogenesis-related genes. However, chlorophyll decomposition is delayed in tropical regions under high temperatures (above 25 °C), and there is no characteristic increase in carotenoids [19]. Therefore, in the present study, the factor governing the delayed colouration under the increased-temperature treatments continued until harvest, which did not promote chlorophyll decomposition. It is thought that the expression of genes related to carotenoids to induce colouration of the pericarp was not proper.

Spiegel-Roy and Goldschmidt [11] mentioned that tropical orange fruits remain mature and marketable only for a short time, after which they rapidly senesce. Li (cited by Zheng et al. [22] also reported that excessive temperature and humidity are likely to cause early plant senescence, shorten the growth period, increase vulnerability to pests and diseases, and reduce fruit yield and quality.

Moon et al. [12] reported that spring heating treatments of Shiranuhi mandarin fruits increased the photosynthesis rate of leaves, which positively affected growth and development. Sucrose and nitrogen have the most important effects on citrus pigmentation during ripening on trees. Nitrogen and sugar are inversely related, because colouration during ripening is induced when the nitrogen content is low and the sugar concentration is high [19]. Therefore, increasing temperatures from spring to harvest promotes vegetative growth such as fruit development, but it is thought that their effects on sugar content, colouration, and yield negatively affect fruit quality and production.

### 3.2. Effects of Excessively High Temperatures on Fruit Taste

Soluble sugars are related to carbon, and amino acids are an intermediate product of nitrogen metabolism. Soluble sugars are known to increase resistance to abiotic stresses [22]. Soluble sugars include mainly glucose, fructose, and sucrose. The amount of sucrose, reaching 15% to 18% of the fresh weight in certain mandarin fruits, exceeds that of fructose and glucose [11]. In our study, the TSS content and the fructose/glucose ratio were lower under the D8 treatment than under the other treatments. Similarly, at the tuber initiation stage of potatoes under elevated CO_2_ and high temperature, the content of hexose (glucose + fructose), which is used as a carbon source, decreased, suggesting that sugar content would decrease if high temperatures increased the rate of metabolism and sugar use [23].

The exposure of fruits on trees to stress and high temperatures during storage was found to be associated with the accumulation of various amino acids associated with glycolysis and the tricarboxylic acid (TCA) cycle [24]. We also found that the markedly increased Gly, Ser, Gln, Asn, and Asp contents contributed to metabolism in relation to the photorespiratory nitrogen cycle under high-temperature treatments. The increased high temperature during the day increased the total amino acid content, but the increased high temperature at night or during the day/night was similar to that of the control. Therefore, it is thought that increased daytime temperatures have a greater influence on the amino acid content than do increased night-time temperatures. Proline (Pro) is known to act as a compatible osmolyte, stabilizing the structure of proteins and ROS scavengers, and accumulating in response to various abiotic stresses [25]. In this study, increasing the high temperature during day or night did not increase the Pro content, but increased high temperature at day/night considerably decreased the Pro content.

Increased daytime/night-time temperature significantly increased the bitterness and sour/umami flavour, while the sweetness and sweetness/bitterness markedly decreased. In the sensory evaluation, the taste of the fruits under the high-temperature treatments was considerably reduced compared to those under the control treatment, with the fruits of the former having a plain or slightly abnormal taste.

Bitterness is well known in grapefruits, but there is almost no bitterness in common mandarin fruits. The bitterness taste of citrus can be classified into two categories: bitterness due to naringin derived from flavonoids, and limonin-based bitterness [26]. However, naringin was not detected in the flavonoid analysis in this study. However, we were unable to analyse the composition of volatile constituents. Therefore, studies on various volatile compounds related to flavour under excessively high temperatures are needed.

The species, cultivar, and genotype of plants have specific optimal temperature ranges for physiological functions that include biosynthesis of secondary metabolites such as phenolics, alkaloids, flavonoids, and terpenoids, and departure from those ranges can affect biomass and biosynthesis of secondary metabolites [27]. In our study, increased daytime temperature decreased the contents of narirutin and hesperidin in the peel, whereas rising night-time temperature increased the narirutin, nobiletin, and tangeretin contents. However, the rising daytime/night-time temperatures caused an increase in only the nobiletin contents. The increase in daytime temperatures led to a decrease in narirutin, hesperidin, and rutin contents, even in the pulp. The effects of increased temperature during the night and during the day/night on the pulp were similar to those of the peel. Kim et al. [28] also showed a change in total flavonoid content in response to low-temperature stress in citrus varieties.

Analysis of the TPC in peel and pulp extracts in response to the different temperature treatments (Table 6) revealed a higher content in the peel than in the pulp. This seemingly occurred because the peel contains more phenolic compounds than the pulp. Sir Elkhatim et al. [29] analysed the total contents of extracts of peels, seed-containing pulp, and whole fruits of three species. The results showed a significant difference between species and that, compared with the pulp and seeds, peels contain phenolic compounds. According to Kim et al. [30], more phenolic compounds are present in the peel than in the pulp, and their contents are higher in non-ripened fruits than in mature fruits.

In conclusion, excessive increases in the temperature during the day, night, or day/night negatively affected fruit size, yield, sugar, acids content, and accumulation of secondary metabolites such as polyphenols and flavonoids, but the accumulation of some amino acids was positively affected. Additionally, the antioxidant activity of ROS scavengers was decreased in response to excessive increases in temperature. Therefore, it is necessary to understand the expression levels of genes related to high temperatures through transcriptome studies, which have recently been performed. In addition, excessive increases in temperature during the day, night, or day/night are expected to negatively affect not only fruit quality but also fruit taste. Consequently, it seems necessary to improve facilities that can minimize damage caused by high temperatures, such as implementing shade screens or developing new varieties with strong high temperature resistance in response to rapidly rising temperatures.

## 4. Materials and Methods

### 4.1. Plant Materials and Excessively High-Temperature Treatments

**Figure 6:**
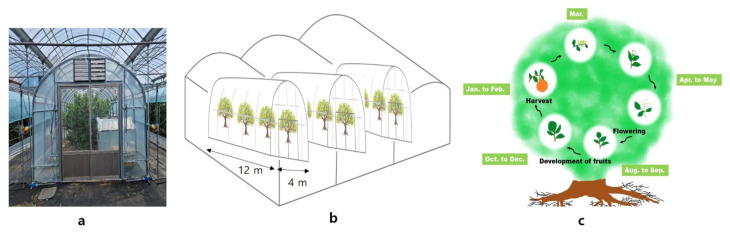
Design of an inner greenhouse with excessively high temperatures within a plastic-film greenhouse and life cycle of Shiranuhi mandarin trees in Korea. (**a**) Individual miniature greenhouse. (**b**) Schematic diagram of miniature greenhouses. (**c**) Life cycle of Shiranuhi mandarin tree.

Nine-year-old Shiranuhi mandarin (*C. unshiu* × *C. sinensis*) × *C. reticulata*) trees grafted onto trifoliate orange, grown in a non-heated plastic-film greenhouse at the Citrus Research Institute, Seogwipo, Korea (elevation of 200 m above sea level, 33°18′06.0″ N, 126°36′39.6″ E), were used as test materials (Figure 6). For the excessively high temperature test, a total of 5 individual miniature greenhouses with individual heaters were installed, and the treatments were imposed via control of the daytime and night-time temperatures. The heating time was from 7:00 a.m. to 7:00 p.m. in the daytime temperature treatments, and 7:00 p.m. to 7:00 a.m. the next day in the night temperature treatments. As shown in Table 7, five different high-temperature treatments were applied: normal atmospheric temperature (Con), provided by open skylight and open side windows; Con + 4 °C during the day (D4); Con + 8 °C during the day (D8); Con + 4 °C during the night (N4); and Con + 4 °C during the day and night (DN4). If the indoor temperature surpassed 42 °C (the maximum allowed temperature), the side windows were designed to open by an automatic system to minimize the damage caused by the excessively high temperature that can occur during hot summers. Temperature treatments were applied from 20 to 270 days after full bloom (DAFB). Fertilizer, irrigation, and pest control practices were performed according to the standard citrus cultivation method of the Rural Development Administration (RDA).

### 4.2. Shoot and Fruit Characteristics

The leaf/fruit ratio, number of shoots, length of shoots, and fruit set were measured at the beginning of July, which was the end of the second physiological drop. Fruit development was evaluated every 2 weeks from 75 to 255 DAFB (the colouration period). Fruit yield was measured after harvest, and the fresh weight of the branches was measured after uniform pruning was performed in early spring.

Fruit width and length were measured using a digital calliper (Mitutoyo Corp., Kawasaki, Japan), and Hunter’s *L*, *a*, and *b* parameters were measured using a CR-400 Chroma meter (Konica Minolta Sensing Inc., Japan) and averaged for three equatorial sites on the fruit surface. Fruit firmness was measured using a TA-XT2 texture analyser (Stable Microsystem Ltd., Surrey, UK) fitted with a 3 mm diameter probe. After measuring each fruit, the rind was removed, and the pulp weight was measured. Each fruit rind was collected and then measured with a calliper. The total soluble solids (TSS) content after separating the pulp and juicing was determined by a refractometer (PAL-1, Atago Co., Ltd., Tokyo, Japan). For acidic content analysis, the samples were diluted five times with distilled water and then titrated with a 0.1 N sodium hydroxide solution in conjunction with two drops of 1% phenolphthalein indicator solution. In addition, the juiced samples were used for free sugar and organic acid content analyses.

### 4.3. High-Performance Liquid Chromatography (HPLC)-Based Analysis of Sugars and Organic Acids

The juice was diluted 10-fold with distilled water and used as a sample. Afterward, 1 mL of each diluted extract was filtered through a 0.2 μm filter. The analysis was carried out using a Prominence ultrafast liquid chromatography (UFLC) system (Shimadzu Co., Ltd., Kyoto, Japan) equipped with a refractive index detector (RID) (RID-20A) for sugars or a UV-visible detector (SPD 20A) for organic acids. Sugars were analysed via a Zorbax NH2 column (4.6 mm × 250 mm × 5 μm, Agilent Technologies Inc., CA, USA). The mobile phase consisted of acetonitrile (ACN)/water (75/25, *v*/*v*); the flow rate was set at 1 mL/min, the column temperature was 30 °C, the injection volume was 10 μL, and the measurement duration was 40 min.

Organic acids were analysed on a Shim-pack GIS C18 column (4.6 mm × 250 mm × 5 μm, Shimadzu Co., Ltd., Kyoto, Japan). In this case, the mobile phase consisted of ACN (solvent B) and 10 mM sodium phosphate solution in water, with a pH of 2.6 (solvent A); the flow rate was 1 mL/min, the column temperature was 30 °C, the injection volume was 10 μL, and measurement duration was 30 min. Gradient elution was initiated at 100% A for 10 min, followed by a gradient elution from 20% A and 80% B for 7 min, to 100% B from 17 to 30 min. All of the samples were extracted and injected in triplicate for UFLC analysis. For quantification, sucrose, fructose, glucose, citric acid, malic acid, and oxalic acid standards (Sigma-Aldrich, St. Louis, MO, USA) were used to generate calibration curves.

### 4.4. Liquid Chromatography-Mass Spectrometry (LC-MS) Analysis of Free Amino Acids

Juice samples were hydrolysed before analysis. For hydrolysis, 100 μL of juice sample was added to 900 μL of methanol (with 0.1% formic acid), after which the mixture was vortexed. After centrifugation at 13,000 rpm for 10 min at 4 °C, the upper layers were filtered through 0.2 μm filters. Free amino acids were analysed using a Waters ACQUITY ultra-performance liquid chromatography (UPLC) system coupled to a tandem quadrupole (Xevo TQ-S) mass spectrometry system via the multiple reaction monitoring (MRM) method. The compounds were separated on an Intrada amino acid column (50 mm × 2 mm × 3 μm, Imtakt). In this case, the mobile phase consisted of ACN/tetrahydrofuran/25 mM ammonium formate/formic acid (9/75/16/0.3) (solvent B) and 100 mM ammonium formate/ACN (80/20, *v*/*v*) (solvent A). The flow rate was 0.4 mL/min, the injection volume was 5 μL, and the measurement duration was 17 min. A gradient elution was initiated at 100% B for 3.0 min, 83% B and 17% A for 6.5 min, 100% A for 10 min, and 100% B for 12–17 min.

### 4.5. HPLC Analysis of Flavonoid Contents and Antioxidant Activity

For the extracts, the peel and pulp were sliced separately, dried at 45 °C for two days and then ground into a powder. Afterward, 30 mL of 70% ethanol was added to 1 g of powder, and the suspension was incubated for approximately 1 h under sonication. The supernatants were then filtered through 0.45 μm polytetrafluoroethylene (PTFE) membrane filters. Each extract was dried in an evaporator at 37 °C, and then dissolved in ethanol/dimethyl sulfoxide (DMSO) (1/1, *v*/*v*). The extracted solution was then used for the measurements of flavonoids, total polyphenols, DPPH and ABTS free radical scavenging activity.

Flavonoids were analysed via HPLC (e2695 Separations Module, Waters Corp., Milford, MA, USA) and a UV/visible detector (Waters 2489, Waters Corp., Milford, MA, USA). The HPLC instrument operational and gradient conditions of the mobile phase were the same as those described by Kim et al. [28].

The total phenolic content (TPC), DPPH free radical scavenging activity, and ABTS free radical scavenging activity were analysed using a spectrophotometer (SpectraMax M2, Molecular Devices LLC., San Jose, CA, USA). All assay conditions were the same as those described by Kim et al. [28]. The TPC was expressed as milligrams per gallic acid equivalents (GAEs), and the 50% inhibitory concentration (IC50) was expressed as the quantity of the extract necessary to react with half of the DPPH or ABTS radicals.

### 4.6. Statistical Analysis

The data were analysed via the R version 3.6.3 software package and the treatment means were separated using a Duncan’s multiple range test (DMRT) in the ANOVA program of R version 3.6.3 software package, with *p* < 0.05, and are expressed as the means ± SEs. All of the determinations were performed at least in triplicate for each sampling.

## Figures and Tables

**Table 1:** Effects of excessively high temperatures on Shiranuhi mandarin shoot growth and fruit harvest in a plastic-film greenhouse.

Treat	Ratio of Leaves to Fruit	No. Shoots/Branch	Length of a Shoot (cm)/Branch	Fruit Set (%) ^a^	No. Fruits/Tree	Total Fruit Weight (Kg)/Tree	Fresh Weight of a Branch (kg)/Tree
Con	98.1 ± 20.7 a ^b^	0.7 ± 0.1 b	26.0 ± 2.3 c	23.3 ± 4.8 ab	130.5 ± 12.9 a	25.9 ± 1.9 a	6.4 ± 0.5 ab
D4	83.3 ± 21.1 a	0.7 ± 0.1 b	26.2 ± 2.8 c	17.1 ± 6.6 ab	103.8 ± 13.1 ab	16.8 ± 4.3 b	5.3 ± 0.9 bc
D8	109.4 ± 31.3 a	0.9 ± 0.0 a	38.0 ± 2.1 ab	12.4 ± 3.9 b	29.5 ± 3.3 d	7.7 ± 0.4 c	8.2 ± 0.6 a
N4	94.3 ± 15.7 a	0.3 ± 0.1 c	43.6 ± 4.9 a	30.9 ± 5.7 a	92.0 ± 7.4 bc	15.5 ± 2.2 b	5.7 ± 0.9 bc
DN4	106.2 ± 22.3 a	0.4 ± 0.1 c	30.3 ± 4.2 bc	12.4 ± 3.1 b	66.0 ± 1.7 c	9.1 ± 1.4 bc	3.7 ± 0.5 c

^a^ The percentage of fruit set was calculated as the number of fruit per number of flowers × 100. ^b^ The data are the means ± SEs (*n* = 16 or *n* = 40). The means followed by different letters within columns are significantly different according to Duncan’s Multiple Range Test (*p* < 0.05).

**Table 2:** Effects of excessively high temperatures on prominent bump on the top of fruit and firmness at harvest time.

Treat	Prominent Bump on the Top of Fruits		Thickness of the Peel (mm)	Firmness (N)
	Diameter (mm)	Length (mm)		
Con	35.5 ± 0.6 c ^a^	16.2 ± 0.7 c	4.8 ± 0.1 a	10.5 ± 0.3 c
D4	39.5 ± 0.6 b	20.7 ± 0.6 b	4.8 ± 0.1 a	10.0 ± 0.2 c
D8	44.1 ± 0.8 a	27.2 ± 2.2 a	5.0 ± 0.1 a	12.8 ± 0.4 a
N4	39.2 ± 0.7 b	20.8 ± 0.9 b	5.0 ± 0.1 a	11.7 ± 0.4 b
DN4	41.1 ± 0.9 b	22.3 ± 0.9 b	4.8 ± 0.1 a	8.6 ± 0.2 d

^a^ The data are the means ± SEs (*n* = 40). The means followed by different letters within columns are significantly different according to Duncan’s Multiple Range Test (*p* < 0.05).

**Table 3:** Effects of excessively high temperatures on the amino acid content (µg/ mL) of fruits of Shiranuhi mandarin trees grown in a plastic-film greenhouse.

	Con	D4	D8	N4	DN4
Gly	4.7 ± 0.4b ^a^	7.4 ± 0.1ab	9.3 ± 1.6a	7.3 ± 1.5ab	6.8 ± 0.5ab
Ala	42.0 ± 2.7a	47.1 ± 5.6a	64.2 ± 19.4a	67.3 ± 9.4a	37.9 ± 4.4a
Ser	66.8 ± 3.6b	81.2 ± 1.4ab	95.3 ± 14.7a	69.3 ± 6.0b	64.4 ± 3.2b
Pro	58.5 ± 1.9a	54.0 ± 8.6ab	57.7 ± 3.9a	53.1 ± 1.5ab	36.0 ± 7.6b
Val	8.5 ± 0.4a	8.7 ± 0.3a	11.2 ± 2.1a	10.0 ± 0.7a	10.0 ± 0.7a
Thr	4.8 ± 0.3c	7.0 ± 0.8ab	6.3 ± 1.0abc	5.4 ± 0.5bc	7.7 ± 0.3a
Leu	1.8 ± 0.1b	1.7 ± 0.1b	2.0 ± 0.3b	2.2 ± 0.2b	3.7 ± 0.5a
Ile	4.5 ± 0.2ab	4.2 ± 0.2b	4.6 ± 0.8ab	5.2 ± 0.4ab	5.8 ± 0.5a
Asn	144.8 ± 6.7b	267.7 ± 26.8a	316.1 ± 24.8a	164.5 ± 25.0b	273.8 ± 21.4a
Asp	146.4 ± 14.8b	176.6 ± 14.1ab	209.1 ± 4.1a	96.2 ± 11.5c	155.1 ± 3.3b
Lys	13.3 ± 0.9a	19.2 ± 3.4a	18.8 ± 5.1a	17.9 ± 1.2a	16.1 ± 1.2a
Gln	58.8 ± 3.3ab	55.1 ± 8.2ab	71.5 ± 9.0a	37.5 ± 4.1b	48.4 ± 7.7ab
Glu	62.6 ± 3.0a	63.6 ± 3.3a	65.8 ± 1.0a	57.9 ± 2.4a	49.9 ± 10.6a
Met	2.3 ± 0.1a	2.5 ± 0.3a	2.9 ± 0.6a	2.7 ± 0.2a	2.6 ± 0.2a
His	4.3 ± 0.4b	5.8 ± 1.3ab	5.3 ± 1.1ab	4.4 ± 0.4b	9.6 ± 2.4a
Phe	11.0 ± 0.8b	14.5 ± 4.2ab	9.7 ± 0.6b	8.9 ± 0.4b	22.3 ± 3.5a
Arg	249.2 ± 9.9a	292.1 ± 21.4a	319.3 ± 93.1a	255.3 ± 21.0a	160.7 ± 63.8a
Tyr	1.1 ± 0.1b	0.7 ± 0.0b	0.8 ± 0.1b	1.4 ± 0.0ab	2.7 ± 1.0a
Trp	2.2 ± 0.4a	3.3 ± 1.6a	6.5 ± 2.3a	2.0 ± 0.1a	6.5 ± 1.7a
Cys	NDb	NDb	NDb	NDb	0.2 ± 0.1a
Total	887.6 ± 24.0b	1112.5 ± 58.8ab	1276.4 ± 159.7a	868.6 ± 71.0b	920.0 ± 69.0b

^a^ The data are the means ± SEs (*n* = 3). The means followed by different letters within columns are significantly different according to Duncan’s Multiple Range Test (*p* < 0.05). ND, not detected.

**Table 4:** Changes in taste characteristics of Shiranuhi mandarin (µg/mL fresh juice) fruits of trees grown under excessively high temperatures.

Treatment	Taste ^a^					
	Sweetness	Bitterness	Sour/Umami	Sweetness/Bitterness	Others	Total
Control	235.7 ± 6.1 ab ^b^ (26%) ^c^	34.5 ± 2.2 b (4%)	353.8 ± 18.3 c (40%)	262.5 ± 10.8 a (30%)	1.1 ± 0.1 b (0.1%)	887.6 ± 24.0 b
D4	251.9 ± 19.9 ab (22%)	40.8 ± 6.5 b (4%)	507.8 ± 37.5 ab (46%)	311.3 ± 22.1 a (28%)	0.7 ± 0.0 b (0.1%)	1112.5 ± 58.8 ab
D8	304.3 ± 36.6 a (24%)	42.2 ± 6.6 b (3%)	591.0 ± 24 a (46%)	338.1 ± 98.2 a (27%)	0.8 ± 0.1 b (0.1%)	1276.4 ± 159.7 a
N4	239.8 ± 13.3 ab (28%)	35.5 ± 2.0 b (4%)	318.6 ± 33.9 c (37%)	273.2 ± 22.0 a (31%)	1.4 ± 0.0 ab (0.2%)	868.6 ± 71.0 b
DN4	201.1 ± 19.4 b (22%)	60.8 ± 8.6 a (7%)	478.8 ± 7.9 b (52%)	176.8 ± 64.8 a (19%)	2.7 ± 1.0 a (0.3%)	920.2 ± 69.0 b

^a^ Sweetness, Ala + Gly + Ser + Thr + Gln + Pro; bitterness, His + Cys + Met + Val + Leu + Ile + Phe + Trp; sour/umami, Asp + Glu + Asn; sweetness/bitterness, Lys + Arg; others, Tyr. ^b^ The data are the means ± SEs (*n* = 3). The means followed by different letters within columns are significantly different according to Duncan’s Multiple Range Test (*p* < 0.05).^c^ The taste ratio to total amino acids.

**Table 5:** Effects of temperature on flavonoid contents (mg/100 g) in the peel and pulp of Shiranuhi mandarin fruits at harvest time.

	Treat	Rutin	Narirutin	Hesperidin	Nobiletin	Tangeretin	Total
Peel	CON	62.1 ± 9.7a ^a^	759.3 ± 69.2ab	2023.6 ± 161.9a	140.3 ± 14.2b	23.5 ± 2.3b	3008.9 ± 228.9a
	D4	44.2 ± 11.6a	552.6 ± 52.0c	1637.3 ± 123.6a	131.2 ± 10.3b	21.3 ± 1.3b	2386.6 ± 176.9b
	D8	45.1 ± 10.8a	504.4 ± 44.3c	1693.1 ± 76.6a	197.2 ± 10.9a	26.3 ± 1.6ab	2466.1 ± 114.9ab
	N4	66.9 ± 11.3a	883.4 ± 78.8a	1819.0 ± 153.0a	202.8 ± 23.5a	31.8 ± 3.4a	3003.9 ± 247.9a
	DN4	61.7 ± 10.2a	584.5 ± 58.0bc	1685.6 ± 104.6a	185.2 ± 13.3a	26.1 ± 1.9ab	2543.0 ± 174.0ab
Pulp	CON	45.5 ± 4.0a	253.4 ± 19.6a	276.8 ± 13.9a	ND	ND	575.7 ± 30.2a
	D4	39.7 ± 2.9a	193.4 ± 8.5bc	235.7 ± 10.7b	ND	ND	468.7 ± 19.0b
	D8	19.7 ± 4.4b	166.2 ± 8.2c	190.3 ± 12.1c	ND	ND	376.1 ± 14.3c
	N4	37.0 ± 2.3a	226.2 ± 10.8ab	211.6 ± 9.0bc	ND	ND	474.8 ± 19.5b
	DN4	39.2 ± 2.8a	211.5 ± 11.7b	187.4 ± 12.1c	ND	ND	438.1 ± 23.7bc

^a^ The data are the means ± SEs (*n* = 8). The means followed by different letters within columns are significantly different according to Duncan’s Multiple Range Test (*p* < 0.05). ND, not detected.

**Table 6:** TPC and antioxidant activity in citrus fruit extracts.

	Treatment	TPC ^a^	DPPH ^b^	ABTS ^b^
Peel	Con	3890.8 ± 132.7 a ^c^	183.3 ± 4.4 b	26.4 ± 0.8 b
	D4	3365.5 ± 114.3 b	227.9 ± 10.5 a	29.3 ± 0.8 a
	D8	3501.6 ± 91.1 b	225.3 ± 13.9 a	27.7 ± 0.5 ab
	N4	3931.9 ± 205.2 a	188.0 ± 12.7 b	28.9 ± 1.4 ab
	DN4	3372.3 ± 70.9 b	213.4 ± 6.8 ab	28.2 ± 0.6 ab
Pulp	Con	1438.0 ± 44.9 a	467.9 ± 21.6 b	107.3 ± 4.4 a
	D4	1355.2 ± 47.6 ab	630.8 ± 38.7 a	113.5 ± 3.0 a
	D8	1074.7 ± 44.3 d	621.8 ± 29.9 a	119.2 ± 7.3 a
	N4	1239.2 ± 39.9 bc	517.9 ± 29.9 b	117.6 ± 4.2 a
	DN4	1213.7 ± 45.8 c	648.0 ± 44.7 a	106.2 ± 3.3 a

^a^ The TPC values are expressed as milligrams of GAE per 100 g of peel or pulp extract. ^b^ IC50, concentration in milligrams per 100 mL required for scavenging 50% DPPH or ABTS radicals. ^c^ The data are the means ± SEs (*n* = 8). The means followed by different letters within columns are significantly different according to Duncan’s Multiple Range Test (*p* < 0.05).

**Table 7:** Temperature treatments and mean temperatures during the experimental period.

Treatment	Time ^a^	Atmospheric Temperature	Mean Temperature Day/Night (°C)
Con	Day/Night	Con	21.4/15.6
D4	Day	Con + 4 °C	25.4/15.5
D8	Day	Con + 8 °C	28.7/15.4
N4	Night	Con + 4 °C	21.7/19.9
DN4	Day/Night	Con + 4 °C	25.6/19.6

^a^ Day, 7:00 a.m. to 7:00 p.m.; night, 7:00 p.m. to 7:00 a.m. the next day; day/night, continuous.

## Data Availability

Not applicable.
